# Complex chemical reaction networks for future information processing

**DOI:** 10.3389/fnins.2024.1379205

**Published:** 2024-03-13

**Authors:** Katja-Sophia Csizi, Emanuel Lörtscher

**Affiliations:** Department of Science of Quantum and Information Technology, IBM Research Europe - Zurich, Rüschlikon, Switzerland

**Keywords:** chemical computing, neuromorphic computing, chemical reaction networks, low-energy, brain-inspired

## Abstract

Tackling the increasing energy demand of our society is one of the key challenges today. With the rise of artificial intelligence, information and communication technologies started to substantially contribute to this alarming trend and therefore necessitate more sustainable approaches for the future. Brain-inspired computing paradigms represent a radically new and potentially more energy-efficient approach for computing that may complement or even replace CMOS in the long term. In this perspective, we elaborate on the concepts and properties of complex chemical reaction networks (CRNs) that may serve as information-processing units based on chemical reactions. The computational capabilities of simpler, oscillatory chemical reactions have already been demonstrated in scenarios ranging from the emulation of Boolean gates to image-processing tasks. CRNs offer higher complexity and larger non-linearity, potentially at lower energy consumption. Key challenges for the successful development of CRN-based computers are associated with their specific physical implementations, operability, and readout modalities. CRNs are sensible to various reaction triggers, and provide multiple and interlinked reaction pathways and a diverse compound space. This bears a high potential to build radically new hardware and software concepts for energy-efficient computing based on neuromorphic architectures—with computing capabilities in real-world applications yet to be demonstrated.

## 1 Introduction

Semiconductor technology constitutes one of the key-enabling technologies responsible for numerous innovations in modern times. Thanks to the continuous miniaturization of all integrated circuit components, the foundational technology has been successfully adapted to varying and diverse computing tasks over several decades. The validity and continuation of Moore's Law is currently being controversially discussed. At the same time, the increasing energy consumption of today's computing infrastructures—combined with other energy-expensive technologies—undoubtedly represents one of the largest challenges to our society. The projected energy demand might soon surpass the amount of energy being cumulatively generated. In the field of information and communication technologies, this is due to an ever increasing number of systems (Internet of Things (IoT), mobile systems, data centers, etc.) but also aggravated by emerging artificial intelligence (AI) applications (image and voice recognition, analog sensor signal processing, chatbots, etc.). The majority of AI applications entails workloads for which the classical von–Neumann architecture, with separated memory and processing units, was originally not intended and now turns out to be costly in terms of energy consumption. Furthermore, maintaining and running AI systems creates similarly high costs as training thereof, currently already consuming >500 MWh per day. Overall, this trend will soon result in unaffordable energy demands beyond 100 TWh if the current systems cannot be substantially improved (de Vries, 2023). Consequently, there are tremendous efforts in semiconductor and related industries aiming at tuning existing semiconductor devices (e.g., phase-change, FPGA), architectures (e.g., specialized architectures), systems (e.g., GPUs, TPUs) or computing tasks (e.g., in-memory computing) for AI applications. Beyond those attempts, more disruptive and radically new ways of computing beyond the use of electrons and transistors are being evaluated. These initiatives include spintronics, quantum computing, optical computing, DNA-based computing, and neuromorphic computing. Generally, it is imperative for all new approaches to prioritize sustainability aspects over the entire life-cycle. This includes the use of abundant materials, green fabrication processes, complete recycling, etc. to contribute to a circular economy. To complement or replace existing technologies, it is essential not only to meet the prevailing standards of scalability and performance, but also to satisfy all aforementioned sustainability constraints. At the moment, there seems to be no obvious successor technology for CMOS. However, neuromorphic architectures appear to be a promising foundation as they conceptually mimic the human brain, which serves as an unparalleled role model in terms of energy efficiency. In addition, some neural networks are already designed from an implicit, simplified brain inspiration, but with orders of magnitude less complexity (Richards et al., 2019; Zador et al., 2023).

In this perspective, we present the novel class of bio-inspired, chemical information processing concepts that are based on complex chemical reaction networks (CRNs). CRNs are capable of processing information based on highly interconnected and interlinked chemical reactions. Due to their chemical self-organization and nonlinear characteristics, these systems provide potentially useful means for low-energy and massively parallel computing. To demonstrate the neuromorphic capabilities, scalable physical implementations and operational protocols must be developed.

## 2 Chemical reactions as information-processing units

The human brain with its interconnected neurons and the release of neurotransmitters in response to nerve impulses across localized information-processing centers is still unmatched in terms of energy efficiency. It consumes only around 20 watts of power while performing more than 200 trillion operations per second. New classes of HPC systems (e.g., ICNS Deep South) parallel such cross-linked brain-inspired architectures and are predicted to reach more than 100 trillion synaptic operations per second at a significant—yet to be measured—energy reduction. The cross-linking and collocation of memory and information-processing units will be at the heart of next-generation, semiconductor-based neuromorphic HPC to solve the von-Neumann bottleneck. Additionally, radically new approaches may take up the information-processing concept of our brain even closer by using chemical compounds and chemical reactions to encode and process information for computing purposes: Not only does information processing on the chemical level in living entities regulate and control fundamental processes like immune response, growth, or gene expression, the human brain runs entirely on chemical reactions for “logic” information processing. It is therefore conceptually appealing to draw direct analogies between the chemical compound and chemical reaction space to bio-inspired brain-type architectures with reactions emulating synapses and compounds representing neurons. By their very nature, molecules can carry out complex tasks such as molecular recognition and chemical reactions with the smallest possible footprint and energy requirements. Furthermore, chemical reactions can be cascaded, and are typically highly non-linear. It has been demonstrated that interconnected chemical systems are capable of mimicking Boolean logic gates (Tsompanas et al., 2021), carrying out pattern recognition (Gizynski and Gorecki, 2017; Parrilla-Gutierrez et al., 2020) or image processing tasks (Rambidi et al., 1998), finding shortest paths (Rambidi and Yakovenchuk, 2001), or solving optimization problems (Guo et al., 2021). Like other non-conventional computing architectures, these attempts predominantly exploited time-dependent event-driven paradigms, either in the form of spike-induced, or self-induced excitations (in analogy to oscillatory, or spiking neural networks).

### 2.1 Beyond Belousov–Zabotinsky reactions

**Figure 1:**
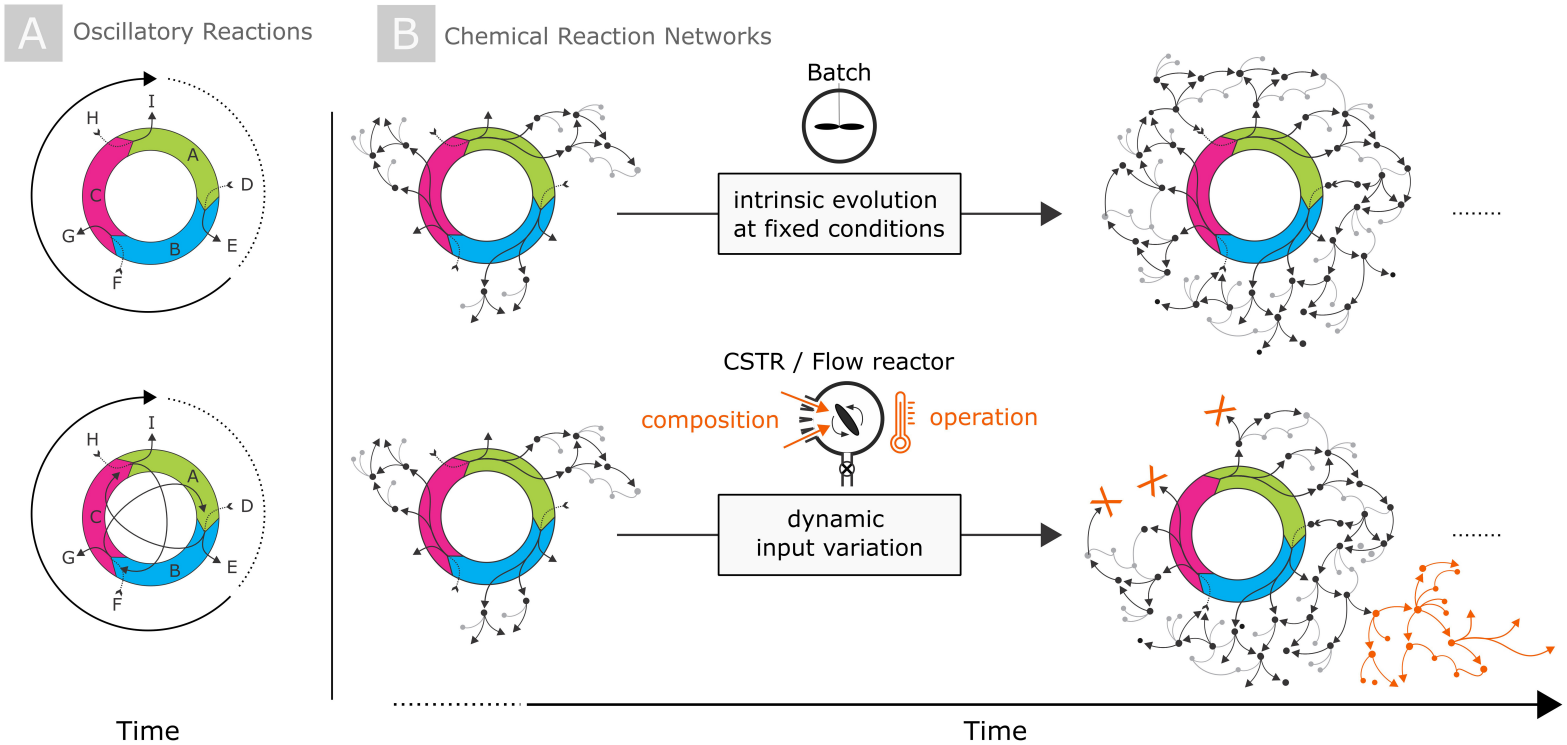
Schematic and highly simplified illustrations of different types of reaction networks: **(A)** The simplest form are closed-loop catalytic cycles as, for instance, occurring in oscillatory reactions including the BZ reaction. In these reaction sequences, either a few dominant intermediates are selectively formed **(top)** or, in more complex scenarios, cross-reactions between intermediates of the main reaction cycle occur **(bottom)**. **(B)** In contrast, CRNs comprise highly-interconnected, cascaded reaction sequences that populate individual branches of the reaction topology over time. The time-evolution of CRNs occurs intrinsically at given fixed environmental conditions **(upper row)**, or can be steered by dynamic input variation of the compositional and/or operational input parameters **(bottom row)**.

Chemical computing was pioneered using the Belousov–Zabotinsky (BZ) reaction. The underlying chemical reactions result in nonlinear temporal oscillations and spatial self-organization. In the BZ oscillator, the time-evolution of excitations is determined by chemical reactions and diffusion, therefore referred to “reaction–diffusion” computing. In a very simplified representation, the BZ can be described by three main reactions that form a closed-loop catalytic cycle, as illustrated in Figure 1A. In BZ oscillations, the clock rate correlates with the intrinsic oscillation frequency and is somewhere between 1 and 100 Hz, not comparable to the GHz frequencies of semiconductor devices (GHz). Apart from that, the aforementioned complexity of information processing in living entities may require massive parallel operation in interlinked compartments. As a potential alternative, chemical reaction networks that have a higher complexity than the BZ reaction have been recently proposed as a chemical computing platform (Ivanov et al., 2023). In principle, any real-world chemical system can be encoded in the form of a chemical reaction network, although the network width and depth (i.e., the number of compounds formed and the number of reactions or reaction sequences that connect these compounds) varies significantly. Figure 1 conceptually illustrates, in a very simplified manner, the different degrees of complexity and interlinkage of chemical reactions suited for computing purposes.

Under the aspect of nonlinearity and complexity, CRNs more closely resemble bio-inspired systems than oscillatory reactions. The prebiotically relevant formose reaction is one of the archetype CRNs of that kind to show a temporal evolution over time once the self-condensation of formaldehyde is energetically overcome. As recently demonstrated, the formose CRN provides a high-dimensional state space, nonlinear interactions, a fading memory effect, and discrete output signals—namely products derived after derivatization of the reaction mixture—that all depend susceptibly on input variations (Robinson et al., 2022). A fading-memory effect can moreover be realized by forcing the CRN into an out-of-equilibrium steady state, where the system can then receive inputs from and adjust its response to environmental conditions by dynamically changing its underlying reactions. With all these properties, the formose CRN can dictate some “design rules” and properties of an artificial CRN to be used for future computing:

1. *Complexity* and *nonlinearity*: The evolution of a reaction network constitutes a highly non-linear self-organization process, as demonstrated for instance by van Duppen et al. (2023) for the formose CRN;
2. *Dynamicity*: This evolution is highly time-dependent, generating complex temporal patterns as a function of different chemical inputs. These patterns can then be modulated in a dynamical way by steering a CRN's steady state through variation of the input parameters;
3. *Parallelizability*: In CRNs, chemical reactions occur simultaneously and independently in a massively parallel manner, realizing the processing of a large amount of information concurrently;
4. *Low-energy operability*: Due to the parallelization and autonomous self-organization capabilities of CRNs, these systems can be operated with extremely low external energy. Furthermore, chemical systems exhibit a propensity to favor pathways associated with the lowest overall system energy (if not steered externally) and therefore autonomously populate the kinetically least constrained reaction pathways;
5. *Determinism and reproducibility*: As chemical reactions are defined by the laws of quantum mechanics, a reaction's outcome and its corresponding rate under given conditions are unequivocally defined and should be precisely predictable and reproducible, following deterministic rules instead of stochastic (random) behavior. However, this does not directly translate to the macroscale operation in a real lab. This is subject to macroscopic effects, diffusion, local concentration effects, evaporation, etc., where the unique but convoluted CRN state must still be characterized by appropriate analytical techniques;
6. *Tunability*: A CRN must be tunable and its properties adaptable to different computing tasks.

**Figure 2:**
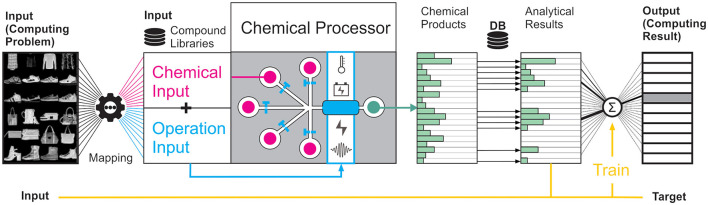
Methodological overview of the principle components and processing steps of a chemical computing platform: Problem-specific data is mapped to chemical (pink) and static as well as dynamic operational (blue) inputs for a given computing problem. These inputs and reaction conditions determine the evolution of the CRN, which acts as an information-processing unit based on the underlying CRN-embedded chemical reaction pathways. The product (or intermediate) space of the CRN (type of compounds and quantities thereof) must be analytically identified and quantified using libraries, and chemometric algorithms. All these characteristic features can be used to train a CRN's response to a desired target function *in-silico*, leading to classification, and forward prediction.

The design of a chemical reaction system that can transmit signals, self-develop at corresponding non-equilibrium conditions and respond to external and internal triggers that affect the evolution to be used in the process of learning, are all crucial aspects when designing a CRN-based computer. Figure 2 depicts the basic components and a simple assembly of a chemical computer based on CRNs. The chemical processor is fed with an operational protocol derived from mapping real-world input data to reaction input parameters, which comprise the initial chemical composition and the reaction starting conditions. The reaction can then be dynamically controlled and steered by changing the chemical input flows and/or the reaction conditions. At different points in time, intermediates and products are formed, which need to be read out by some type of analytical instrument to collect output signals. For details and challenges associated with encoding and read-out, see Section 2.3.

### 2.2 Hardware requirements and challenges for physical implementation

Compared to CMOS architectures, characterized by its deterministic and scalable circuit design (rules), 3D assembly and hierarchies with device and redistribution layers, wiring schemes, thermal management and so forth, chemical reactions naturally occur in a liquid environment and are often not solid state in nature. Conceptually, computation of arbitrary complexity has been theoretically demonstrated to be Turing complete in principle, even by using only a small number of different molecular species (Soloveichik et al., 2008). This can be achieved by storing and processing information as integer counts of molecules in a well-mixed solution. If chemical systems are perceived as stochastic, the error probability is reduced at each computing step, and the total error probability can made arbitrarily small by adjusting the initial molecular species count. Then, a stochastic CRN can solve any computational problem—no matter how complex—given enough time and memory. However, a physical implementation of any chemical computing approach into real-world computing devices and systems will represent a disruptive change in design, fabrication and operation compared to existing semiconductor architectures with the following fundamental questions yet to be addressed:

1. Can automation and suitable hardware provide sufficient control over all chemical reactions to reproducibly create identical output states—both qualitative and quantitative—of a CRN?
2. Do CRNs behave chaotically or do their reaction pathways follow certain rules?
3. Can CRNs be cascaded to enable a scalable computing platform?
4. How fast can the system be encoded and what is the typical latency?
5. How can chemical reactions be fueled as reagents are being consumed?
6. What are means to clock a CRN?
7. Does self-organization and self-limitation within a CRN scale or is there any fundamental limitation when miniaturizing it?

An obvious approach to handle and govern control over wet-chemistry is to compartmentalize chemical reactions, e.g., by introducing physical reactor volumes, following similar strategies as found in biological systems and used when the required selectivity cannot be achieved (Ruiz-Mirazo et al., 2014). In that sense, semiconductor architectures and fabrication processes can be highly beneficial as they enable scalable reaction volumes down to *fL* with great flexibility regarding reactor volumes and types (static reactors vs. flow reactors), while offering a high chemical resistivity against corrosive solvents. In addition, the implementation of smallest channels for mass-flow, and ion- and proton-selective materials such as membranes is feasible. Furthermore, microfluidic platforms provide means to control the reaction (e.g., dwell time, temperature, etc.) and to monitor and feedback-control it, for instance through electrode implementation. Highly complex 3D liquid networks can be envisioned that may enable site-selective supply of materials, e.g., to locally feed reactions or to steer the reaction by providing reagents. The precise supply of feedstock molecules is a crucial aspect in chemical computing, as chemical compounds are consumed over time and must be fed for long term operation. Currently, the lifetime of a chemical processor is limited to a few minutes to hours, depending on the CRN's kinetics. Furthermore, silicon-based microfluidics may enable a seamless integration into a CMOS stack or the direct use of CMOS components suitable for controlling and monitoring wet-chemical systems.

For a proof-of-concept, the chemical computer may still be operated manually, involving typical labor-intensive chemical procedures. For repeated use, unavoidable when processing larger data sets, efficient operation can only be achieved if the platform can be operated in full automation, ensuring reproducibility and scriptability of all components including in-line analytical readout, and *in silico* inference. In particular for CRNs where the composition must be very accurately controlled at any time, only a script-based orchestration of liquid handling hardware, reactor operation and analytics can provide a precision suitable for achieving reproducible chemical operations. In addition, handling and disposal of chemicals require compliance with various safety standards. Furthermore, the safe operation and risk assessment relies on prior knowledge and understanding of the intermediates and products formed. Another critical aspect is the realizable computational speed: The typical latency of the CRN, together with intrinsic kinetic properties that determine the reaction speed, can only be modulated to a certain degree, and therefore constitute a severe computing bottleneck. Furthermore, the determination of the CRN state might require time-intensive post-processing steps, e.g., derivatization, separation, etc. to characterize the products both quantitatively and qualitatively. All these parameters are by no means trivial to predict, and must be empirically addressed in time-consuming parameteric studies when designing a new CRN for computing.

### 2.3 Encoding and readout

Beyond the design and operation of the chemical processor itself, a real-world computing task must be encoded into the chemical world, and the corresponding solution decoded from the properties derived from the chemical system. For that purpose, specific problem-related data is mapped onto a typically rather sparse input subspace, which comprises the chemical composition and the operational input parameters at which the CRN can autonomously self-develop. The non-equilibrium conditions that span this subspace, however, are generally challenging to predict *a priori* and require time-intensive parametric studies of this multidimensional space. To obtain solutions to a given computing problem, suitable analytical methods are required to read out the complex state of the CRN which is in principle given by the type of products and their concentrations. In case the response and temporal evolution of the CRN is not yet known for the entire parameter space (chemical input + reaction conditions + initial state), high-resolution analytics must be employed to identify and quantify all compounds. These methods are most often based on sample extraction, preparation, separation and physio-chemical sensing modalities, thereby creating a speed bottleneck for computing. Suitable instrumentation includes gas or liquid chromatography, mass spectrometry, trapped-ion mobility, differential ion mobility etc., many of them further need to be combined to achieve a complete picture. All these offline methods cannot directly be incorporated into the computing platform itself due to size limitations and an interruption of the chemical reaction process upon sample extraction. In return, compounds are identifiable through comparison with huge libraries, supported by chemometric algorithms. These quantifications, which can be traced down to parts-per-trillion levels, enable reasoning of the detailed behavior of a CRN, at the cost of speed and ease of operation.

At a later stage with known CRN behavior, such methods are not suitable any more. Instead, a lower analytical resolution is sufficient to determine essential features of the CRN state. Suitable inline/online methods enable direct sensing within the reactor or integration of the sensing component into the computing platform to enable automated and timely measurements without interrupting or terminating the platform operation. These methods comprise, for instance, pH and electrochemical potential measurements, ultraviolet—visible or Fourier-transformed infrared (FTIR) spectroscopy and benefit from real-time data acquisition. Mostly, however, quantitative information is presented in highly convoluted features. For instance, in FTIR spectroscopy, features may be assigned to characteristic functional groups, and the total absorption intensities can be integrated, but not dissected into individual compound's quantities. However, as long as enough of such features are present in the spectra – which is the case for CRNs with high chemical diversity in their output space – the signals represent accumulated concentrations, which are directly proportional to the individual compounds, thereby still representing the CRN state. Furthermore, the analytics may only identify a subset of all compounds generated or properties measured in an even more convoluted way, including for instance (spectrally broad) absorption or emission features, which are still cumulative properties of the analytical matrix. In principle, only state-representative, essential features are required to determine the state of the CRN. Hence fully untargeted fingerprinting may be another reasonable analytical modality for computing at much less experimental effort. Consequently and by its nature, any reasoning of the CRN's chemical composition and behavior will then not be possible anymore but the simpler readout may be better scalable, cheaper and faster while still providing enough features for computing. Hence, all these considerations constitute a trade-off between precision, resolution, speed and ease of use.

The exact timing and sequence of sampling of the CRN state by analytical means is not trivial to assess, and the nature of the data acquisition determines the scope of application. While measurements of the instant response of the CRN are only limited by the speed of instrumental data acquisition, monitoring of an equilibrium or steady state depends on the inherent chemical kinetics that govern reactions and that will lead to the formation of new chemical species. These kinetics dictate a specific evolution time for each type of CRN to unfold its complexity. Subsequently, a single CRN state can be sampled, yielding already enough data for classification tasks. In contrast, the CRN reservoir state can be modulated by static or dynamic changes in the input concentrations too, providing a timely response of the network to evolve and develop under these variations. This allows for monitoring a time-resolved read-out of the CRN state. This data can be leveraged for various time-dependent computing tasks, such as forward prediction, modeling complex dynamics of biological systems, or solving voice recognition tasks, as the time-dependent CRN input and output can always be mapped to these types of problems.

### 2.4 Potential application areas

With the chemical computing platform depicted in Figure 2, various applications, both in chemical sciences but also general computing, can be envisioned. Conceptually, the CRN can be considered a material embodiment of a fixed and non-linear type of reservoir, whose properties are considered a black box as being unknown at the beginning. For small networks, the behavior of the reservoir may be mimicked by these AI/Machine Learning algorithms, whose structure and dynamic behavior are explainable. In reservoir computing, input variables are mapped to the dynamics of a fixed system called a reservoir, whose response is then read out by determining its state and mapped to the desired computing solutions. For CRNs, there are multiple features that may represent the state. A major advantage of the reservoir computing approach for CRNs is the comparatively low training effort as weights connecting reservoir nodes do not need to be assigned explicitly, but are chosen randomly such that only the readout-layer is trained. In a CRN, the intermediates and reaction paths connecting these intermediates must hence not be characterized explicitly, which would require the derivation of reaction rate constants. However, if the chemical behavior of the CRN is explainable, it can be encoded as a graph in which compound nodes are connected by weights derived from reaction kinetics, and must not be treated as a black box. However, the mapping of a CRN reservoir‘s input layer to product output data generates interpretable input–output correlations. These can be used to perform simple classification or optimization tasks, for instance the maximization of product outputs in the chemical discovery sector. In a more long-term vision, they could even be harnessed for the *in-situ* synthesis of drug molecules for personalized patient treatment in the health-care sector. This makes CRNs ideal candidates to forecast the spatiotemporal behavior of dynamic and even chaotic systems.

## 3 Discussion

The energy-related economic and societal boundary conditions imposed by an ever-increasing energy demand fuel the innovative pressure to design fundamentally new computing approaches. In this perspective, we discussed one emergent, brain-inspired computing paradigm that exploits complex chemical reaction networks as information processing units. Chemical reaction networks are highly nonlinear, energy-efficient, and parallelizable and therefore capable to mimic the information processing capabilities of living systems, whose computing efficiency is still unparalleled. However, an actual physical implementation of a chemical (reservoir) computer poses various challenges associated with operability, encoding of a real-world computing problem, and readout. These operational parameters must be addressed carefully, as they constitute a disruptive design change compared to the predominant semiconductor architectures. Achieving a profound understanding of the time-dependent behavior of complex chemical reaction networks will enable the comprehension of biological reaction networks and help improve automated and yield-optimized retrosynthesis with multiple applications not efficiently tackled by today's computing systems.

## Author contributions

K-SC: Conceptualization, Methodology, Visualization, Writing – original draft, Writing – review & editing. EL: Conceptualization, Funding acquisition, Project administration, Resources, Supervision, Visualization, Writing – original draft, Writing – review & editing.
